# Osteoprotegerin induction in response to microbial infection

**DOI:** 10.1186/ar3668

**Published:** 2012-02-09

**Authors:** Yasunari Takada, Koichi Matsuo

**Affiliations:** 1 Laboratory of Cell and Tissue Biology, Keio Univ. School of Medicine, Tokyo 160-8582 Japan

## 

Osteoprotegeirn (OPG) is an endogenous decoy receptor for RANKL, which is a cytokine essential for osteoclast differentiation. Lipopolysaccharide (LPS) is known to induce osteoclast formation when injected onto calvaria in mice. Unexpectedly, we observed that mice injected with LPS up-regulate OPG and down-regulate RANKL levels in peripheral blood.

In the present study, we examined whether OPG is induced by microbial infection of various kinds, and the sites and significance of OPG production in infected mice. Wild-type mice infected with*Salmonella*, *Staphylococcus*, *Mycobacteria*or influenza virus showed increase in OPG levels in peripheral blood. We also found that the levels of OPG in serum of human patients infected with *M. tuberculosis *and *M. avium *were significantly increased. Moreover, injection of mice with LPS induced OPG production specifically in lymph nodes, especially in high endothelial venule (HEV)cells, but not in other organs. OPG production was suppressed in c-Fos-deficient mice and enhanced in Fra-1 transgenic mice, indicating that OPG production is regulated by AP-1 transcription factors. Loss of OPG in mice did not affect either their survival or *Salmonella *proliferation in spleen and liver after infection with virulent strains of *Salmonella*. Interestingly, however, when wild-type mice were infected with an avirulent* Salmonella *strain, which can induce OPG, osteoclast development was suppressed and bone mineral density was increased. These data reveal for the first time that lymph nodes protect bones from infection-induced bone loss through OPG production.

